# Tooth-Ache

**Published:** 1899-12

**Authors:** Constantine Hering


### TOOTH-ACHE.

CONSTANTINE HERING, M. D.

### LOCATION OF PAINS.

**Most in the front-teeth,** Bell., Caust., Carbo-veg., Cham., China, Coff., Ign., Merc., *Nat-mur.*, *Nux-mos.*, *Nux-v.*, Phos., Phos-ac., *Rhus*, Sil., Staph., *Sul.*

**Most in the Eye and Stomach-teeth,** Acon., Calc., Hyosc., *Rhus*, Staph.

**Most in the Molars or Back-teeth,** Arn., Bell., *Bry.*, Calc., *Carbo-veg.*, Caust., Cham., China, Coff., Hyosc., Ign., Merc., Nux-mos., Nux-v., *Phos.*, Phos-ac., Puls., Rhus, Sil., *Staph.*, Sul.

**Most in the Upper-teeth,** *Bell.*, Bry., *Calc.*, *Carbo-veg.*, *China*, *Nat-mur.*, *Phos.*

**Most in the Lower-teeth,** *Arn.*, *Bell.*, Bry., Carbo-veg., *Caust.*, *Cham.*, China, Hyosc., Ign., Merc., Nux-v., Phos., Puls., *Rhus*, *Sil.*, *Staph.*

**One-sided,** Acon., Bell., Cham., Merc., Nux-v., Puls.

**On the Left side,** Acon., Apium-v., Arn., Carbo-veg., Caust., *Cham.*, China, Hyosc., Merc., *Nux-mos.*, *Phos.*, Rhus, Sil., *Sul.*

**On the Right side,** *Bell.*, Bry., Calc., Coff., Lach., Nat-mur., Nux-v., Phos-ac., Staph.

**A whole Row of Teeth,** Cham., Merc., Rhus, Staph.

**In Hollow Teeth,** *Ant-crud.*, *Bell.*, Bry., *Calc.*, Carbo-veg., Caust., *Cham.*, China, Coff., Hepar, *Hyosc.*, *Lach.*, *Merc.*, Nux-mos., Nux-v., Phos., Phos-ac., *Puls.*, *Rhus*, Sil., *Staph.*, Sul.

**In the Gums,** Ant-crud., Arn., *Bell.*, Bry., *Calc.*, *Carbo-veg.*, Cham., China, Hepar, Hyosc., Lach., *Merc.*, *Nat-mur.*, Nux-mos., *Nux-v.*, Phos., Phos-ac., Puls., Rhus, Sil., *Staph.*, Sul.

— **Upper,** Bell., *Calc.*, Nat-mur.

— **Lower,** Caust., Phos., *Staph.*, Sul.

— **Interior of,** Arn., Nat-mur., Phos-ac., Puls., Rhus, *Staph.*

### OBJECTIVE SYMPTOMS.

**In the Gums, Swollen**, Acon., Bell., Calc., Cham., Carbo-veg., Caust., China, Hepar, Lach., Nux-v., Nat-mur., Phos., Puls., Rhus, Sul.

— **Painful**, Apium-v., Arsen., Calc., Carbo-veg., Caust., Lach., Merc., Nux-mos., Nux-v., Phos., Staph., Sul.

— **Bleeding**, Bell., Calc., Carbo-veg., Caust., Lach., Merc., Nux-mos., Nux-v., Phos., Staph., Sul.

— **Ulcerated**, Bell., Calc., Carbo-veg., Caust., Hepar, Lach., Merc., Nat-mur., Nux-v., Phos., Staph., Sil.

### CHARACTER OF THE PAINS.

**Pressing**, Acon., *Arn.*, Bry., Carbo-veg., Caust., China, Hyosc., Ign., Nat-mur., Nux-mos., Nux-v., Phos., *Rhus*, Sil., *Staph.*, Sul.

— **Inwards**, Rhus, Staph.

— **Outward**, Phos.

— **Asunder**, Phos-ac.

— **As if from Congestion of the Blood, as if the teeth were too close**, Acon., Arn., Bell., Cham., Calc., China, *Coff.*, Hepar, Hyosc., *Nux-v.*, *Puls.*

**As if pulled out or wrenched**, Arn., Caust., Nux-mos., Nux-v., Phos-ac., Rhus.

**Too Long**, Arn., Arsen., Bell., *Bry.*, Calc., Carbo-veg., *Caust.*, Cham., Lach., Hyosc., Nat-mur., Nux-v., Rhus, Sil., *Sul.*

**Loose**, Arn., Arsen., Bry., Carbo-veg., Caust., Cham., China, Hepar, *Hyosc.*, Ign., Merc., Nat-mur., Nux-mos., Nux-v., Phos., Puls., Rhus., Staph., Sul.

**As if too Loose**, Arsen., Bry., *Hyosc.*, Merc., Rhus.

**Blunt**, Acon., China, Dulc., Ign., Lach., Merc., Nat-mur., *Nux-mos.*, Phos., Phos-ac., *Puls.*, Sil., Staph., Sul.

**Sore, Bruised**, Arn., Arsen., Bell., Bry., Calc., Carbo-veg., Caust., *Ign.*, Nat-mur., Nux-v., Phos., *Puls.*, *Rhus.*

**Burning**, Cham., Merc., Nat-mur., Nux-v., Phos., Puls., Rhus, Sil., Sul.

**Gnawing, Scraping**, Cham., Nux-v., Rhus., Staph.

**Digging**, Ant-crud., Bry., Calc., China, Ign.

**Boring**, Bell., Calc., Lach., Merc., Nat-mur., *Nux-v.*, Phos., Phos-ac., *Sil.*, *Sul.*

**Jerking, Twitching**, Apium-v., Anti-cru., Arsen., Bry., Bell., Calc., Caust., Cepa, Cham., Coff., Hepar, Hyosc., Lach., *Merc.*, *Nux-v.*, *Puls.*, *Rhus*, *Sul.*

**Drawing, Tearing**, Ant-crud., Bell., Bry., Carbo-veg., Calc., Cepa, Cham., China, Glon., Hyosc., Lach., Merc., Nux-v., Phos-ac., Rhus, Staph.

**Cutting, Piercing**, Acon., Ant-crud., Bell., *Bry.*, Calc., Caust., *Cham.*, *China*, Lach., *Merc.*, Nux-v., Nux-mos., Phos., Phos-ac., *Puls.*, Rhus, Sil., Staph.

**Beating, Pulsating**, *Acon.*, Arn., Arsen., *Bell.*, Calc., Caust., Cham., China, Coff., Glon., *Hyosc.*, Lach., Merc., Nat-mur., Phos., Puls., Rhus, *Staph.*, Sul.

**Intermittent**, Bell., Bry., Cham., Coff., Calc., China, Merc., Nux-v., Puls., Rhus, Sil., Staph., Sul.

### CAUSE.

**Caused by damp night-air**, Nux-m.

— **Damp-air**, Merc.

— **Cold damp weather**, Nux-m., Cepa, Rhus.

— **Wind**, Acon., Puls., Rhus, Sil.

— **Draught**, Bell., Calc., *China*, Sul.

— **Taking Cold**, Acon., *Bell.*, *Bry.*, Calc., Caust., Cham., China, Coff., *Dulc.*, *Hyosc.*, Ign., *Merc.*, *Nux-v.*, *Nux-m.*, *Phos.*, *Puls.*, *Rhus*, Staph., Sul.

— **Taking Cold, when overheated**, Glon., Rhus.

— **by getting wet**, Bell., Calc., Caust., Hepar, Lach., Nux-m., Phos., Puls., Rhus, Sul.

— **Suppressed Perspiration**, Cham., Rhus.

### AGGRAVATION.

**Constant, day and night**, Bell., Calc., Caust., Nat-m., Sil., Sul.

**During the day only, better in the night**, Merc.

— **none in the night**, Calc., Bell., Merc., Nux-v.

— **worse in bed**, Ant-c., Merc.

**Worse in the night**, Acon., Ant-c., Ars., *Bell.*, Bry., *Carbo-v.*, *Cham.*, China, Coff., Hepar., Hyosc., Merc., Nat-m., Nux-m., Nux-v., Phos., Phos-ac., *Puls.*, *Rhus*, Sil., *Staph.*, *Sul.*

**By night only, not during the day**, Phos.

**Most before midnight**, Bry., *Cham.*, China, Nat-m., Rhus, Sul.

— **after midnight**, Ars., Bell., Bry., Carbo-v., Cham., China, *Merc.*, Nat-m., Puls., Phos., Rhus, *Staph.*, Sulph.

**When awaking**, Bell., Carbo-v., Lach., Nux-v. (See Sleep.)

**In the morning**, Ars., Bell., Bry., Caust., Carbo-v., China, *Hyosc.*, Ign., Nat-m., *Nux-v.*, Phos., Phos-ac., Puls., *Rhus*, *Staph.*, Sul.

**At noon**, Cocc., Rhus.

**Afternoon**, Calc., Caust., Merc., *Nux-v.*, Phos., *Puls.*, Sul.

**Towards evening**, Puls.

**At night**, Ant-c., *Bell.*, Bry., Calc., Caust., Hepar, *Hyosc.*, Ign., *Merc.*, Nux-m., Nux-v., *Phos.*, *Puls.*, *Rhus*, Staph., *Sul.*

**Every other day**, China, Nat-m.

**Every seventh day**, Ars., Phos., Sul.

**In spring**, Acon., Bell., Bry., Calc., Carbo-v., Dulc., Lach., Nat-m., Nux-v., *Puls.*, Rhus, Sil., Sul.

**In summer**, Ant-c., Bell., Bry., Calc., Carbo-v., Cham., Lach., Nat-m., Nux-v., Puls.

**In autumn**, Bry., China, Merc., Nux-v., Nux-m., Rhus.

**In winter**, Acon., Ars., Bell., Bry., Calc., Carbo-v., Caust., Cham., Dulc., Hepar, Hyosc., Ign., Merc., Nux-m., *Nux-v.*, Phos., Phos-ac., Puls., *Rhus*, Sil., Sul.

**Getting worse from Cold air**, Bell., Calc., Hyosc., Merc., Nux-m., Nux-v., Sil., Staph., Sul.

— **in the mouth**, Acon., *Bell.*, Bry., Calc., Caust., Hyosc., *Merc.*, Nux-m., Nux-v., Phos., Puls., Sil., Staph., Sul.

— **Opening of the mouth**, Bry., Cham., Caust., Hepar, Nux-v., Phos., Puls.

— **Breathing**, Puls.

— **Drawing air into the Mouth**, *Ant-c.*, Bell., Bry., Calc., Caust., Hepar, Merc., Nat-m., Nux-m., Phos., Sil., Staph., Sul.

**Getting worse from Cold washing**, Ant-c., Bry., *Calc.*, Cham., *Merc.*, *Nux-m.*, Nux-v., Puls., Rhus, Sil., Staph., Sul.

— **Eating cold things**, Bry., Calc., Cham., Nux-v., Puls., Rhus, Staph., Sul.

— **Drinking cold things**, Bry., Calc., Caust., Cham., Hepar, Lach., Merc., Nat-m., Nux-m., *Nux-v.*, Puls., Sil., Staph., Sul.

— **Rinsing of the Mouth with Cold Water**, Sul.

— **Cold in general**, *Ars.*, Ant-c., Calc., Carbo-v., Merc., Nat-m., Nux-m., Nux-v., *Puls.*, Phos-ac., *Rhus*, Sil., Staph., Sul.

**In the open air**, *Bell.*, Calc., Caust., *Cham.*, *China*, Hyosc., Merc., *Nux-m.*, *Nux-v.*, *Phos.*, Puls., *Rhus*, *Staph.*, Sul.

— **Staying**, Bell., Bry., Cham., Hyosc., Merc., Nux-v., Phos-ac., Staph., Sul.

— **Walking**, Nux-v., Phos., *Staph.*

**In a Room**, Apium-v., Ant-c., *Cham.*, Hepar, Nux-v., *Puls.*, Sul.

— **after coming out of the open air**, Phos.

**In a warm Room**, Bry., Cepa, Cham., Hepar, Nux-v., *Puls.*, Phos-ac.

**Warm Stove**, Ars., Puls.

**External warmth**, Bry., Cham., Hepar, Merc., Nux-m., Nux-v., Phos., Phos-ac., Puls., Rhus, Staph., Sul.

**Something warm**, Bry., *Calc.*, Carbo-v., Cham., Coff., Lach., Merc., Nat-m., Nux-v., Phos-ac., *Puls.*, Sil., Sul.

**Eating warm things**, Bry., Calc., Cham., Nux-v., Phos., Puls., Sil.

**Something hot**, Bell., *Calc.*, Phos-ac.

**Drinking warm things**, *Bry.*, Cham., Lach., Merc., Nux-m., Nux-v., Puls., Rhus, Sil.

**Warm bed**, Bel., Bry., Cham., Merc., Nux-v., Phos., Phos-ac., *Puls.*, Rhus.

**Getting warm in bed**, Cham., Merc., Phos-ac., Phos., Puls.

**Drinking**, Cham., Calc., Caust., *Lach.*, Merc., Puls., Rhus, Sil.

**Cold or warm**, Lach.

— **Water**, Bry., Calc., Carbo-veg., Cham., Merc., Nux-v., Puls., Sil., Staph., Sul.

— **Wine**, Aconi., Ign. [Nux-v., *after wine*].

**Getting worse from Drinking Malt liquors,** Nux-v., Rhus.
— **Coffee,** Bell., Carbo-veg., Cham., Coccul., Ign., Merc., Nux-v., Puls., Rhus.
— **Tea,** China, Coff., Ign., Lach.
**Smoking tobacco,** Bry., Cham., China, *Ign.*, Merc., Nux-v.
**Salty things,** Carbo-veg.
**Eating**, Ant-crud., Arn., Bell., Bry., Calc., *Carbo-veg.*, *Caust.*, *Cham.*, Coccul., Hepar, Hyosc., Lach., *Merc.*, Nux-m., Nux-v., *Phos.*, Phos-ac., *Puls.*, Rhus, Sil., *Staph.*, Sul.
**Only while eating,** Coccul.
**After eating,** Ant-c., *Bell.*, *Bry.*, Calc., Cham., China, Coff., *Ign.*, Lach., *Merc.*, *Nat-m.*, *Nux-v.*, Rhus, *Staph.*, *Sul.*
**Chewing,** Arn., Ars., Bell., Bry., Carbo-veg., Caust., *China*, Coccul., Coff., *Hyosc.*, *Ign.*, *Merc.*, *Nat-m.*, *Nux-v.*, Phos., Phos-ac., Puls., Sil., Staph., Sul.
**Only while chewing,** China.
**Swallowing,** Staph.
**Biting,** Ars., Bell., Bry., Calc., Carbo-veg., Caust., China, Coff., *Hepar*, Hyosc., Lach., *Merc.*, Nux-v., Phos., Phos-ac., Puls., *Rhus*, Sil., Staph., Sul.
— **something soft,** Verat.
— **soft food,** Coccul.
— **hard food,** Merc.
**Touched by the food,** Bell., Ign., Nux-v., Phos., Staph.
**Picking,** Puls.
**Cleaning,** Carbo-veg., Lach., Phos-ac., Staph.
**Touching,** Ant-c., Arn., Ars., *Bell.*, *Bry.*, Calc., *Carbo-veg.*, Caust., *China*, Coff., *Hepar*, Ign., *Merc.*, Nat-m., Nux-m. *Nux-v.*, Phos., *Puls.*, *Rhus*, *Sul.*, *Staph.*
— **with the tongue,** Carbo-veg., China, Ign., Merc., Phos., Rhus.
— **even very softly.** *Bell.*, Ign., Nux-v., Staph.
**Pressing on the teeth,** Caust., China, Hyosc., Nat-m., Staph., Sul.
**Sucking the gums,** Bell., Carbo-veg., Nux-m., Nux-v., Sil.
**Rising,** Ign., Merc., Plat.
**Moving the body,** Arn., *Bell.*, *Bry.*, China, *Merc.*, Nux-v., Phos., Staph.

**Getting worse from Moving the mouth**, Caust., Cham., Merc., Nux-v.

**Talking**, Nux-m.

**Deep breathing**, Nux-v.

**Being at rest**, Ars., Bry., Cham., Puls., Rhus, Staph., Sul.

**Sitting**, Ant-c., Merc., *Puls.*, *Rhus.*

**— too much**, Acon.

**While lying down**, *Ars.*, Bell., Bry., *Cham.*, Hyosc., Ign., Merc., Nux-v., Phos., *Puls.*, *Rhus*, Staph., Sul.

**— on the painful side**, Ars., Nux-v.

**— on the painless side**, Bry., Cham., Ign., Puls.

**— in bed**, Bry., Cham., Nux-v., Puls.

**In bed**, *Ant-c.*, Bell., Bry., *Cham.*, *Merc.*, Nux-v., Phos., *Puls.*

**Sleep with yawning**, Staph.

**When going to sleep**, Ant-c., Ars., Merc., Sul.

**While asleep**, Merc.

**When awaking**, Bell., Bry., Calc., Carbo-veg., Lach., Nux-v., *Phos.*, Sil., *Sul.*

**Mental emotions**, Acon.

**Vexation**, Acon., Cham., Rhus, Staph.

**Passion**, Nux-v.

**Mental exertions**, Bell., Ign., Nux-v.

**Reading**, Ign., Nux-v.

**Noise**, Calc.

**Being talked to by others**, Ars., Bry.

**Menstruation, before**, Ars.

**— during**, Calc., *Cham.*, Carbo-veg., Nat-m., Lach., Phos.

**— after**, Bry., Calc., Cham., Phos.

**During pregnancy**, Apium-v., Bell., Bry., Calc., Hyosc., Merc., Nux-m., Nux-v., Puls., Rhus, Staph.

**While nursing**, Acon., Ars., Bell., Calc., China, Dulc., Merc., Nux-v., Phos., Staph, Sul.

### SUITABLE FOR.

**For women**, Acon., Apium-v., Bell., Calc., Cham., China, Coff., Hyosc., Ign., Nux-m., Puls.

**For children,** Acon., *Ant-c.*, Bell., Calc., Cham., Coff., Ign., Merc., Nux-m., Puls., Sil.

**For irritable, nervous persons,** Acon., Bell., Cham., Coff., China, Hyos., Nux-m.

**For persons who have taken much Mercury,** Bell., Carb-v., Hep., Lach., Staph.

**— who drink much coffee,** Bell., Carb-v., Cham., Cocc., Merc., Nux-v., Puls., Sil.

### AMELIORATION.

**Getting better from—**

**Cold air,** Nux-v., Puls.

**Wind,** Calc.

**Uncovering,** *Puls.*

**Drawing air into the mouth,** Nux-v., *Puls.*

**Cold washing,** Bell., Bry., Cham., Puls.

**External cold,** Bell., Bry., Cham., China, Merc., Nux-v., Phos., *Puls.*, Staph., Sulph.

**Cold hand,** Rhus.

**Finger wet with cold water,** Cham.

**Holding cold water in the mouth,** Bry., Cepa, Coff.

**Cold drinking,** Bell., Bry., Cham., Merc., Nux-v., Phos., Puls., Rhus, Sulph.

**In the open air,** Ant-c., Bry., Cepa, Hep., Nux-v., *Puls.*

**In the room,** Nux-v., Phosph., Sulph.

**External warmth,** Ars., Bell., Calc., Cham., China, Hyosc., Lach., Merc., Nux-m., Nux-v., Puls., *Rhus*, *Staph.*, Sulph.

**Wrapping up the head,** Nux-v., Phos., Sil.

**Eating something warm,** Ars., Bry., Nux-m., Nux-v., Rhus, Sulph.

**Drinking something warm,** Nux-m., Nux-v., Puls., Rhus, Sulph.

**Getting warm in bed,** Bry., Nux-v.

**Drinking,** Bell.

**Smoking tobacco,** Merc.

**When eating,** Bell., Bry., Cham., Phos-ac., Sil.

**After eating,** Arn., Calc., Cham., Phos-ac., Rhus, Sil.

**When chewing,** Bry., China, Coff.

**Getting better from biting**, Ars., Bry., China, Coff.
**Picking the teeth, so that they bleed**, Bell.
**Picking the teeth**, Phos-ac.
**Rubbing the teeth**, Merc., Phos.
**Touching the teeth**, Bry., Nux-v.
**Sucking the gums**, Caust.
**Pressing upon the teeth**, Bell., China, Bry., Ign., Nat-m., Puls., Phos., Rhus.
**Moving**, *Puls.*, *Rhus.*
**When walking**, Puls., Rhus.
**When at rest**, Bry., Nux-v., Staph.
**Sitting up in bed**, Ars., Merc., Rhus.
**Getting up**, Phos., Nux-v.
**When lying down**, *Bry.*, Merc., *Nux-v.*
**— on the painful side**, *Bry.*, Ign., Puls.
**— painless side**, Nux-v.
**— lying down in bed**, Merc., Puls.
**In bed**, Sulph.
**When going to sleep**, Merc.
**After sleep**, Nux-v., Puls.

### EXTENSION OF PAINS.

**The pains extend**
- **to the jawbones and face**, Lach., Merc., Nux-v., Hyos., Rhus, Sulph.
- **to the cheeks**, Cham., Caust., Bry., Merc., Sil., Staph., Sulph.
- **into the ears**, Ars., Bry., Calc., Cham., Hepar., Lach., Merc., Staph., Sulph.
- **into the eyes**, Caust., Cham., Merc., Puls., Staph., Sulph.
- **into the head**, Ant-c., Ars., Cham., Hyos., Merc., Nux-v., Rhus, Staph., Sulph.

### ACCOMPANIMENTS.

**With headache**, Apium-vir., Glon., Lach.
**— rush of blood to the head**, Acon., Calc., China, Hyos., Lach., Puls.

**With swollen veins**, of the forehead and hands, China.
— **heat in the head**, Acon., Hyos., Puls.
— **burning of the eyes**, Bell.
— **flushed cheeks**, Acon., Arn., Bell., Cham., Merc., Nux-m., Nux-v., Phos., Puls., Rhus, Sulph.
— **pale face**, Acon., Arsen., Ign., Puls., Staph., Sulph.
— **swelling of the cheek**, Arn., Ars., Bell., Bry., *Cham.*, Lach., *Merc.*, Nat-m., Nux-v., Puls., Phos., Phos-ac., Staph., Sulph.
— **salivation**, Bell., Dulc., Merc.
— **dry mouth and thirst**, China.
— **dry mouth without thirst**, Puls.
— **dry throat and thirst**, Bell.
— **chilliness**, Puls., Rhus.
— **heat**, Hyos., Rhus.
— **warm perspiration**, Hyos.
— **chilliness, heat, thirst**, Lach.
— **diarrhœa**, Cham., Coff., Dulc., Rhus.
— **constipation**, Bry., Merc., Nux-v., Staph.

---

